# Oxidative Stress Gene Expression Profile Correlates with Cancer Patient Poor Prognosis: Identification of Crucial Pathways Might Select Novel Therapeutic Approaches

**DOI:** 10.1155/2017/2597581

**Published:** 2017-07-09

**Authors:** Alessandra Leone, Maria Serena Roca, Chiara Ciardiello, Susan Costantini, Alfredo Budillon

**Affiliations:** Experimental Pharmacology Unit, Istituto Nazionale Tumori Fondazione G. Pascale-IRCCS, Naples, Italy

## Abstract

The role of altered redox status and high reactive oxygen species (ROS) is still controversial in cancer development and progression. Intracellular levels of ROS are elevated in cancer cells suggesting a role in cancer initiation and progression; on the contrary, ROS elevated levels may induce programmed cell death and have been associated with cancer suppression. Thus, it is crucial to consider the double-face of ROS, for novel therapeutic strategies targeting redox regulatory mechanisms. In this review, in order to derive cancer-type specific oxidative stress genes' profile and their potential prognostic role, we integrated a publicly available oxidative stress gene signature with patient survival data from the Cancer Genome Atlas database. Overall, we found several genes statistically significant associated with poor prognosis in the examined six tumor types. Among them, FoxM1 and thioredoxin reductase1 expression showed the same pattern in four out of six cancers, suggesting their specific critical role in cancer-related oxidative stress adaptation. Our analysis also unveiled an enriched cellular network, highlighting specific pathways, in which many genes are strictly correlated. Finally, we discussed novel findings on the correlation between oxidative stress and cancer stem cells in order to define those pathways to be prioritized in drug development.

## 1. Introduction

Reactive oxygen species (ROS) are commonly identified as oxygen reactive molecules associated with a wide variety of physiologic events [1] as well as cancer, diabetes, obesity, neurodegeneration, and other age-related diseases [2, 3]. A reduction-oxidation (redox) reaction concerns the transfer of electrons (reducing power) from a more reduced (nucleophilic) to more oxidized (electrophilic) molecules. ROS can be classified in two groups: (1) free radical ROS containing one or more unpaired electron(s) in their outer molecular orbitals (i.e., superoxide radicals and hydroxyl radicals); (2) nonradical ROS which are chemically reactive and can be converted to radical ROS (i.e., hydrogen peroxide), although they do not have unpaired electron(s). In both cases, ROS can be produced by either enzymatic reactions (i.e., NADPH oxidase, metabolic enzymes such as the cytochrome P450 enzymes, lipoxygenase, and cyclooxygenase) or by nonenzymatic reactions, such as during the mitochondrial respiratory chain. These considerations highlight the concept that the source of ROS is extremely heterogeneous. Indeed, ROS can be found in the environment, as pollutants, tobacco smoke, and iron salts, or generated inside the cells through multiple mechanisms [4]. Within cells, mitochondria, cytosol, single membrane-bound organelles (peroxisomes, endosomes, and phagosomes), or exosomes shed from plasma membranes, as well as extracellular fluids, including plasma, are all involved in ROS generation [3, 5, 6]. Mitochondria are the main ROS producers, principally because they are the site of the respiratory chain when electron leakage can react with molecular oxygen, resulting in the formation of superoxide, which can subsequently be converted to other ROS molecules. Then, generated ROS either can be detoxified or can leave the organelle through channels such as voltage-dependent anion channels (VDAC) or aquaporin, or by small vesicles such as exosomes [3, 5, 7]. However, ROS can also be the product of *β*-oxidation in peroxisomes, of prostaglandin synthesis and detoxification reactions by cytochrome P450, or of NADPH-mediated reaction in phagocytes [4, 5].

**Figure 1:**
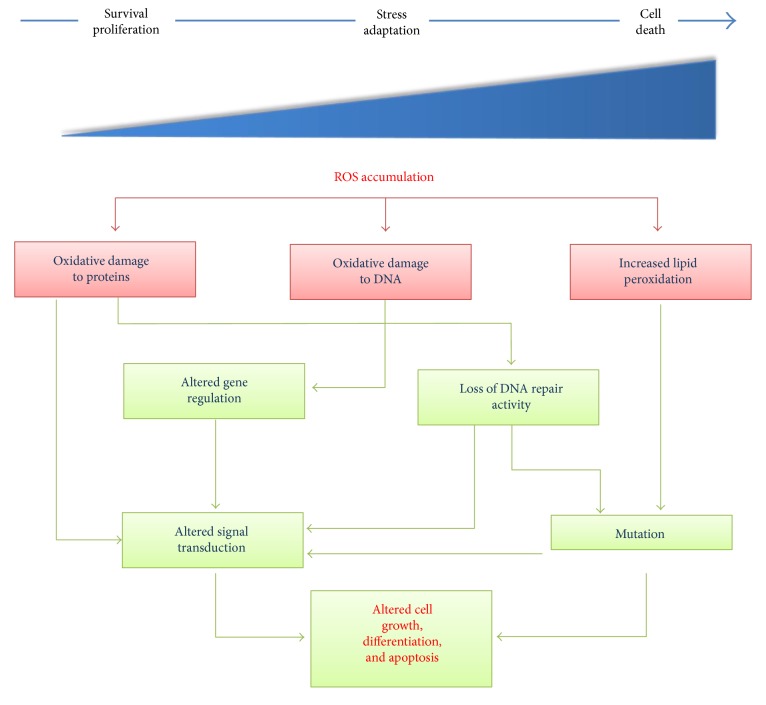
Redox stress activation in physiology. The production of abnormally large amounts of ROS leads to persistent changes in signal transduction and gene expression that, in the last instance, could give to cell death. The steady-state levels of ROS are determined by the rate of ROS production and their clearance by scavenging mechanisms.

ROS are biologically important in a variety of physiological systems, including adaptation to hypoxia, regulation of autophagy, immunity, differentiation, and longevity. They regulate many signal transduction pathways by directly reacting with proteins and by modulating transcription factors and gene expression [1]. At low levels, ROS promote cellular proliferation, differentiation, and migration as well as cellular stress-responsive survival pathways such as nuclear factor-*κ*B (NF-*κ*B), thus inducing proinflammatory cytokines [4, 8]. Because of ROS' highly reactive potential toward biological molecules, excessive ROS levels can damage cellular components such as DNA, proteins, and lipids. To counteract these effects, cells activate “ROS adaption” mechanisms, involving several antioxidant ROS scavengers, as glutathione peroxidase (GPx), thioredoxin (Trx), catalase (CAT), superoxide-dismutase (SOD), and the nuclear factor erythroid 2 (NRF2) pathway [4, 7]. If a further increase in ROS levels occurs, then the cells undergo apoptotic cell death (Figure 1). Therefore, under physiological conditions, in order to guarantee cellular redox homeostasis, cells regulated intracellular ROS levels by applying a tight regulation of ROS generation and of ROS detoxifying pathways.

In this review, we first summarized the role of oxidative stress molecules in cancer initiation and progression and the proposed oxidative stress-targeted anticancer approaches. Next, in order to derive cancer-type specific oxidative stress gene profiles and their potential prognostic role, we integrated a publicly available oxidative stress gene signature [9] with the data extracted from the Cancer Genome Atlas (TCGA) database. Then, we reviewed some of those genes/pathways correlating with patient's survival, in order to define potential novel anticancer therapeutic targets. Finally, we highlighted novel findings on the correlation between oxidative stress and cancer stem cells (CSC).

## 2. The Role of Oxidative Stress Molecules in Cancer Initiation and Progression

A link between ROS and cancer progression dates back to 1981 when increased levels of H_2_O_2,_ induced by insulin were shown to promote tumor cell proliferation. Almost three decades later, several studies sustained this hypothesis, reporting increased levels of oxidative damage products in clinical tumor specimens and plasma as well as in cancer cell lines [5]. Based on these evidences, to date, the idea that altered redox balance and deregulated redox signaling are strongly implicated in any steps of carcinogenesis as well as in the resistance to treatment, by affecting many, if not all, hallmarks of cancer is widely accepted [10, 11]. Indeed, currently, the role of ROS in cancer initiation and progression through the modulation of cell proliferation, apoptosis, angiogenesis, and the alteration of the migration/invasion program is well described [7, 12, 13]. For example, ROS may affect proliferation by a ligand-independent transactivation of different receptor tyrosine kinase via ERK activation and may induce tissue invasion and metastatic dissemination by activation of metalloproteinases. Moreover, the release of vascular endothelial growth factor and angiopoietin induced by ROS promote tumor angiogenesis and *anoikis* [12, 14].

Nonetheless, the exact origin of ROS generation during cancer development and disease progression and how this event could be druggable remains still unclear. Increasing evidences reported a link between ROS activation and the presence of some oncogenes, such as Ras, c-Myc, or Bcr-Abl [2, 15, 16]. Activation of oncogenic signaling might contribute to the increase of ROS levels, which in turn by promoting genomic instability could affect both nuclear and mitochondrial DNA. The consequent activation of antioxidants' signaling within tumor cells can also promote cancer progression and metastasis [2, 15–18]. Furthermore, cancer cells undergo metabolic changes to counteract the oxidative stress, also contributing to metastatic program [5, 19, 20].

Loss of functional p53 is involved in ROS induction, due to p53 “genome guardian” role in sensing and removing oxidative damage to DNA, thus preventing genetic instability [5, 21]. Anyhow, unlike oncogenes, the role of tumor suppressors in the modulation of ROS is more complex, depending on the specific tumor suppressor itself. For example, ataxia-telangiectasia mutated (ATM) is a cellular damage sensor that by regulating cell cycle and DNA repair preserves genomic integrity. Deficiency of ATM gene, either in patients or in mice, has been shown to produce elevated ROS levels and a chronic oxidative stress status. Recently, cytoplasmic ATM is described to activate a pathway leading to autophagy through repression of mammalian target of rapamycin complex 1 (mTORC1) in response to elevated ROS levels [22, 23]. Another example regards the loss of PTEN that determines AKT hyperactivation and inactivation of the forkhead homeobox type O (FoxO) transcription factor and therefore enhanced susceptibility to oxidative stress [24].

Less evidences are available about the regulation of ROS by microenvironment; however, new efforts have been recently focused in this field [5, 12]. In this regard, Chan et al. demonstrated that cancer-associated fibroblast- (CAF-) derived ROS are able to induce the acquisition of an oxidative CAF-like state on normal fibroblasts. Then, these oxidatively transformed normal fibroblasts promoted the development of aggressive tumors via a TGF*β*1-mediated Smad3 signaling, suggesting an important relationship between the extracellular redox state and cancer aggressiveness [25].

## 3. Targeting Oxidative Stress as Anticancer Therapy

The first approach to prevent or treat cancer, by targeting ROS, was based on the use of antioxidant reagents [11, 15]. In one of the first trials, based on supplementation of selenium, vitamin E and *β*-carotene on the diet showed a reduction of overall mortality and cancer rates [26]. However, a following trial not only failed to obtain consistent results but also indicated that in certain cases, antioxidants can rather promote cancer initiation and progression. Concordantly, two trials of cancer prevention, the CARET on male smokers, treated with vitamin A and/or *β*-carotene and the SELECT trial, on older males treated with vitamin E and/or selenium, resulted in an increased incidence of lung and prostate tumors, respectively [27–29]. Similar contradictory results were shown in the trials using antioxidant treatment as adjuvant therapy [30].

Based on these results, almost a decade ago, ROS inducers were proposed as anticancer strategy, in order to overcome the specific threshold of ROS level beyond which cancer cells undergo ROS-mediated cell death [4, 5]. The first agents used are those improving electrons leak from the respiratory complexes in the mitochondria, such as the arsenic trioxide, or conventional chemotherapeutic drugs such as doxorubicin. Indeed, patients treated with those agents showed lipid peroxidation in their plasma as well as low levels of vitamin E, vitamin C, and *β*-carotene in the blood [4]. The mechanism of action of these agents seems to be related to their ability to generate ROS directly from the mitochondria. Indeed, doxorubicin and arsenic trioxide penetrate in the inner membrane of the mitochondria and induce superoxide radical production by modulating the electron transport chain. Also 5-fluorouracil increases mitochondrial ROS with a different mechanism, mediated by p53 [4, 31]. Ionizing radiations represent other important ROS inducers, because they are able to promote by themselves high level of ROS and also because they might increase NADPH oxidase, an important source of ROS [32]. Moreover, we and others have demonstrated, in different models and in different combination settings, that oxidative injury played a significant functional role in the antitumor effect of histone deacetylase inhibitors (HDACi), a class of epigenetic antitumor compounds currently in clinical practice in haematological malignancies [7, 13, 33–42].

Recently, a new ROS inducer compound, Elesclomol (STA-4783), has been developed and tested, both in in vitro and in vivo preclinical studies as well as in clinical trials [5, 43]. Interestingly, the result from a phase II trial using Elesclomol in combination with chemotherapy, in malignant melanoma patients, showed ROS generation and oxidative damage associated with prolonged progression-free survival [44]. Unfortunately, these results were not replicated in a phase III trial, where Elesclomol treatment was suspended due to adverse toxic effects [45]. The reason of this failure could be ascribed, at least in part, to cancer cells' capability to activate ROS adaption mechanisms by increasing levels of ROS scavengers, especially at advanced stages. This event is particularly efficacious in CSC, as described in the last paragraph of this review. To counteract the ROS adaptation mechanisms, a plausible solution could be the combination of ROS inducers either with another ROS inducer or with compounds that suppress cellular antioxidants, to overcome the threshold useful to induce cell death, The latest approach was tested by using an inhibitor of the scavenger SOD2, 2-Me, in combination with arsenic trioxide in lymphocytic leukemia and urothelial carcinoma cells [46, 47]. Similarly, the combination between the inhibitor of the antiapoptotic protein bcl2 ABT-737 and the ROS inducer, N-(4-hydroxyphenyl) retinamide, or the combination between an NRF2 inhibitor and a glutathione-depleting agents, showed increasing therapeutic efficiency compared to single-agent treatment [48, 49]. Based on these data, several clinical trials of combination treatment between ROS inducers and scavenger inhibitors are ongoing, including a multicenter phase II trial with the iron chelator Triapine and gemcitabine in advanced non-small-cell lung cancer [5].

## 4. Bioinformatics Correlation between Oxidative Stress Gene Expression and Prognosis in Solid Cancer Patients

**Figure 2:**
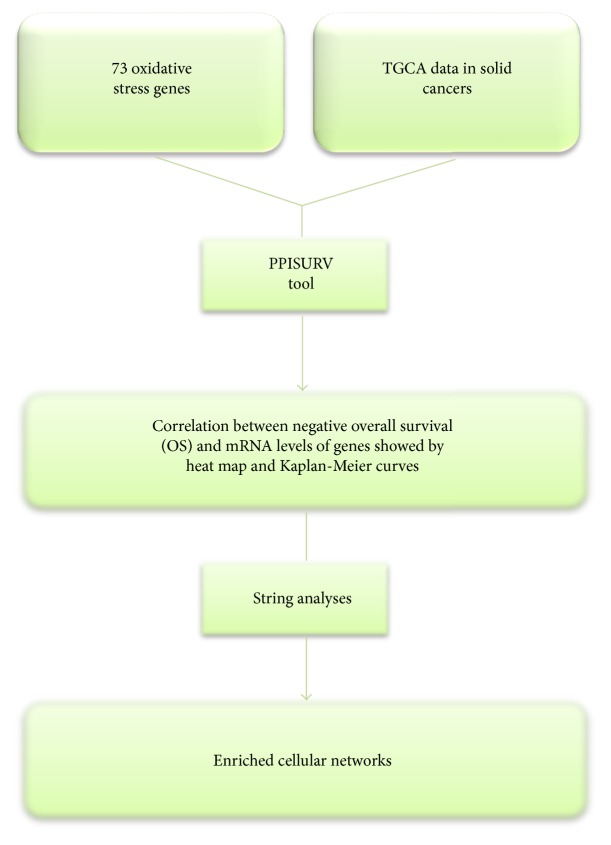
Bioinformatics analyses. Flow chart reporting step-by-step bioinformatics approach to unveil the most important genes/pathways involved in the correlation between oxidative stress and cancer.

Although the biological role of oxidative stress pathways has been extensively demonstrated, it is still unclear which and how oxidative stress genes predict bad prognosis and if their modulation is cancer-type specific. Here, to address this question, we took advantage of Cancer Genome Atlas (TCGA) database that, by profiling RNA expression levels and DNA mutational status for thousands of genes, has generated comprehensive maps of the key genomic changes in several types of cancer, enabling correlative analysis of critical cellular pathways involved in each type of cancers [50, 51]. In details, we compared cancer patient overall survival (OS) and the mRNA levels of 73 oxidative stress genes, selected from a public available oxidative stress signature [9], in different solid tumors. Specifically, the signature included peroxidases, which are represented by glutathione peroxidases (GPx) and peroxiredoxins (TPx); genes implicated in ROS metabolism (i.e., DUSP1, FoxM1, and HMOX1); and genes involved in superoxide metabolism, such as superoxide dismutase (SOD). Starting from the selection of the 73 oxidative stress genes, bioinformatics investigations were performed as described in Figure 2.

In details, bioinformatics analysis was made by SynTarget online tool (http://www.bioprofiling.de/PPISURV) using the following public datasets: TCGA_PAAD for pancreatic cancer, TCGA_COAD for colon cancer, TCGA_HNSCC for head and neck cancer (HNSCC), GSE31210 for lung cancer, TCGA_PRAD for prostate cancer, and METABRIC for breast cancer [52, 53]. PPISURV automatically derives the currently known interactome for a gene of interest and correlates expression levels of its interactome, with survival outcome in multiple publicly available clinical expression data sets containing microarray expression data set annotated with survival data. In details, as reported by Antonov et al. [54], in the case of the option “single gene survival analyses on a single data set,” the PPISURV program exploits rank information from expression data sets that reflect the relative mRNA expression level. The samples are grouped with respect to expression rank of the gene in order to correlate survival information to the expression level of a gene in a particular data set. The groups are then subdivided in basis to “low expression” and “high expression” where expression rank of the gene is less or more than average expression rank across the data set, respectively. This separation of patients into “low” and “high” groups in the data set along with survival information is then used to find any statistical differences in survival outcome and to draw Kaplan-Meier plot. Hence, PPISURV establishes a correlation of the selected gene with survival and assesses the sign of the effect and if the gene deregulation is associated with positive or negative outcome.

**Figure 3:**
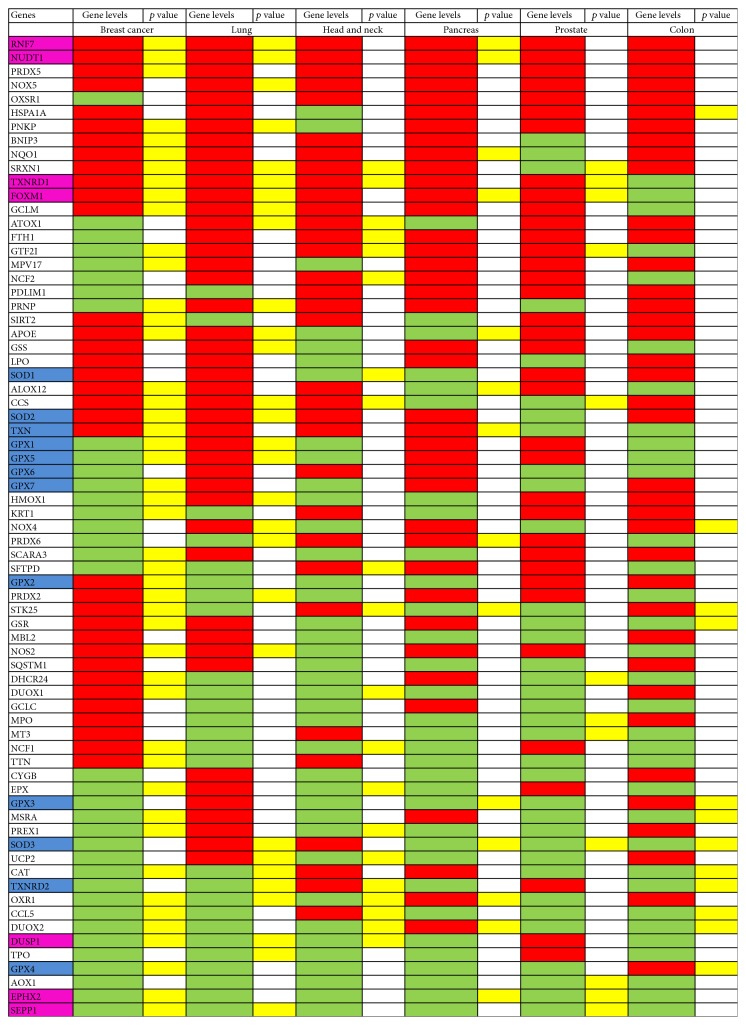
Bioinformatics correlation between oxidative stress gene expressions and poor prognosis in 6 different tumor types. Heat map in which we report in red or in green if the high or low expression of genes was negatively correlated with survival, respectively. Moreover, we evidenced in yellow when the correlation is statistically significant (with *p* value <0.05). In the first column, we evidenced in magenta, the genes similar modulated among cancers and in blue those oxidative stress family extracted from STRING analysis. Notably TXNRD1 showed a central role in both analyses.

Notably, a significant number of oxidative stress genes were negatively correlated with survival in solid carcinomas, reinforced the idea that oxidative stress plays a crucial role in cancer cells (Figure 3). Furthermore, going deep to our bioinformatics analysis, we observed that breast, lung, and HNSCC cancers were those more susceptible to oxidative stress gene expression fluctuations. To explain these data, one hypothesis could be that all these tumors are more vulnerable to external insults (i.e., pollutants) that, as mentioned above, are an important source of ROS. Furthermore, we speculate that this phenomenon could be also related to the high mutational load of those tumors. Indeed, several studies showed that either breast (particularly triple-negative subgroup) or lung cancer exhibited an elevated mutational load which is closely associated to mutations in DNA damage repair genes as well as to intrinsic genomic instability [55–57]. Similarly, recently it has been demonstrated that the overall mutational load was higher in old HNSCC patients that represent a high percentage of all HNSCC cancers, compared to younger patients [58]. On the contrary, pancreatic, prostate, and even colon (with exception of microsatellite instability (MSI) high subgroup) cancers are described as less hypermutated and thus, we speculate, are also less dependent to the oxidative stress and genomic instability [59–61].

**Figure 4:**
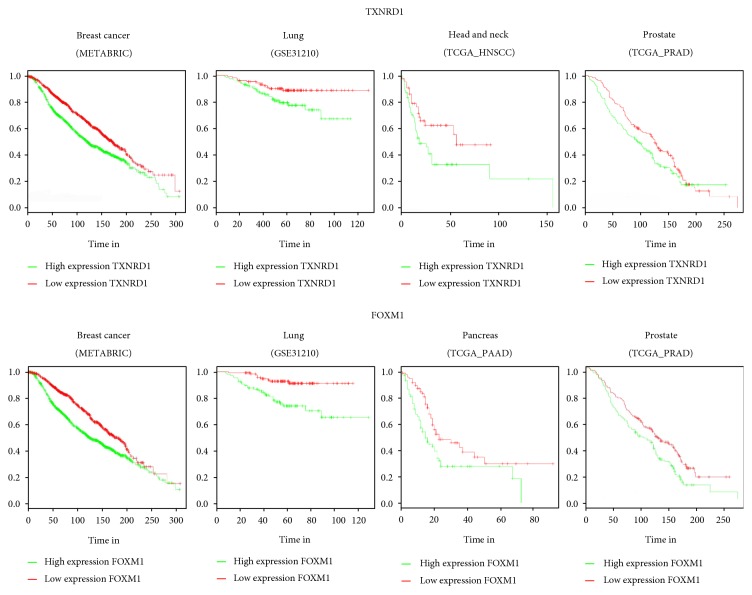
TXNRD1 and FoxM1 expression related to patient survival. Kaplan-Meier curves showing the survival in the case of high and low expression of TXNRD1 and FOXM1 in solid cancer patients.

A further detailed analysis of our correlation between oxidative gene expression signature and OS unveiled that the behavior of modulated genes was different among the cancers examined, with the exception of two genes involved in ROS metabolism, such as FoxM1 and TXNRD1, found as statistically significantly high in poor prognosis patients in four out of six of the tumor types analyzed (Figures 3 and 4). For this reason, those two genes are described below in details in two specific sections of the review. Other five genes, DUSP1, EPHX2, NUDT1, RNF7, and SEPP1, demonstrated a statistically significant modulation in poor prognosis patients in three out of six tumor types (Figure 3 and Suppl. Figure S1 available online at https://doi.org/10.1155/2017/2597581). Briefly, DUSP1 is a dual-specificity phosphatase-1, which is recognized as a key player for inactivating different MAPK isoforms. Recently, a role of DUSP-1 as central redox-sensitive regulator in monocytes has been demonstrated [62]. EPHX2 is a cytosolic epoxide hydrolase, implied in cancer progression and metastasis, in differentially manner based on the stages of carcinogenesis. Indeed, Bracalante et al. demonstrated that in A7 melanotic cells, resembling less aggressive tumor cells, anti-oxidant genes, including EPHX2, were upregulated in response to oxidative stress, while they were downregulated in G10 metastatic melanoma cells [63]. NUDT1, nudix hydrolase 1, is the most prominent mammalian enzyme among other enzymes responsible for hydrolyzing oxidized DNA precursors. NUDT1 is commonly upregulated in a wide variety of tumors to avoid incorporation of oxidized nucleotides that, in turn, induce DNA damage and cell death [64]. RNF7 (RING finger protein-7) acts as a metal chelating protein, a scavenger of ROS at the expense of self-oligomerization. RNF7 was found overexpressed in several tumor types, especially in lung carcinoma, and associated with poor prognosis [65].

SEPP1 is a selenoprotein 1, involved in cellular incorporation of the selenium circulating in the plasma. Moreover, SEPP1 has some antioxidant activity, as target of NRF2 family. In agreement, Bae et al. showed that some antioxidant genes known also as NRF2 targets, including SEPP1, were also transcriptionally modulated by the oncosuppressor BRCA1, thus suggesting that BRCA1 regulates the activity of NRF2 and protects cells against oxidative stress [66].

**Figure 5:**
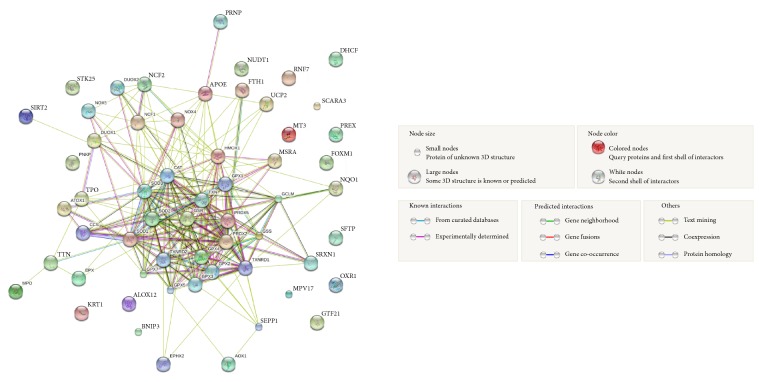
STRING analysis of the 58 oxidative stress genes. Association network in STRING analysis shows interactions of glutathione peroxidase, superoxide dismutase, and thioredoxin as principal oxidative stress signaling among six different tumor types.

Finally, in order to identify a more relevant oxidative stress family in our setting, we performed an additional bioinformatics analysis where, independently from their trend of expression associated to poor prognosis, all modulated genes were analyzed in the biological database STRING, a resource of known and predicted protein-protein interaction. As shown in Figure 5, our analysis reveals an enriched cellular network, in which many genes, including GPx, SOD, and Trx pathways (the latter including TXNRD1), are strongly correlated, as demonstrated by both experimental studies and text mining (see red and green lines, resp.). Similar analyses were also performed for each tumor type separately, or considering high or low gene expression individually, confirming in almost all tumor types GPx, SOD, and Trx signaling as those predominant (Suppl. Figures S2 and S3).

Based on these analyses, together with TXNRD1, we decided to review the correlated pathways enriched in the network, in details (see below), analyzing their role in cancer and the possible therapeutic strategies to hit them.

## 5. FoxM1, a Critical Regulator of Oxidative Stress during Tumorigenesis

The highly conserved transcription factor FoxM1 belongs to the forkhead box transcription factor family, similarly to the best known member of FoxO family. However, different from the members of FoxO family, FoxM1 is expressed only in proliferative cells. Indeed, FoxM1 as a target of the cyclinD-CDK4/6 kinases, is reactivated when quiescent cells reenter in the cell cycle and reach a maximal level in S-phase which is maintained throughout G2 and mitosis [67, 68]. Beyond this role on proliferation, FoxM1 regulates metastasis, apoptosis, and DNA damage repair [69–71]. Furthermore, FoxM1 has been shown to prevent oxidative stress-dependent premature senescence. Park et al. showed how ROS themselves are inducers of FoxM1 expression, which in turn is able to stimulate antioxidant genes. The authors proposed the inhibition of FoxM1 as a new therapeutic strategy to kill cancer cells selectively [71]. In agreement, FoxM1 knocking-down was reported to sensitize human pluripotent stem cells to oxidative stress, as a consequence of activated-CAT5 downregulation, a FoxM1 antioxidant target gene [69].

A growing body of evidences reported high FoxM1 as frequently related to poor prognosis in multiple cancers, concordantly with our bioinformatic results [50]. To date, several mechanisms have been proposed to explain the activity of FoxM1 in cancer progression, including the activation of FoxM1 by several oncogenic protein and signalling pathways, such as c-Myc, Ras, and PI3K/AKT [72].

Hereafter, we discussed the role of FoxM1 in the four tumor types where we found statistically significant association of FoxM1 expression and poor prognosis.

The impact of FoxM1 in breast cancer progression is widely demonstrated. Indeed, its high level has been correlated with large tumor size, lymphovascular invasion, lymphnode metastases, and high stage. Two independent studies carried out on ER+ patients, reported that low FoxM1 expression, compared to high FoxM1 expression, is associated to better survival. Another study proved a positive correlation between HER2 status and FoxM1 expression in breast cancer tissue compared to normal breast counterpart [73–75], suggesting that FoxM1 is a downstream target of HER2 and could be used as a marker of HER2 overexpression. However, molecular basis underling the described roles of FoxM1 in cancer progression still needs to be clarified and different mechanisms have been proposed. For example, the induction of EMT by activation of Slug [76], stabilization of Smad3/Smad4 complex, and activation of TGF*β* pathway [77] as well as the modulation of extracellular matrix by affecting the levels of uPA, uPAR, MMP-2, MMP-9, and VEGF have been proposed [78, 79]. Moreover, FoxM1 cooperates with survivin and nuclear XIAP in the promotion of chemoresistance [80]. Finally, further studies demonstrated that FoxM1 induces resistance to all the therapeutics tested in breast cancer (including cisplatin, paclitaxel, and trastuzumab) by several mechanisms: (1) acting on DNA-damage repair pathways, (2) promotion of cell cycle progression, (3) inhibition of cell cycle checkpoints, and (4) apoptosis induction [72].

FoxM1 gene is widely described as amplified also in lung cancer, regulating cell proliferation by promoting both G1/S and G2/M transition, differentiation, and transformation [81] as well as inhibition of apoptosis [82]. Recently, a direct link between FoxM1-induced ROS and lung cancer progression has been proposed by Tahmasbpoura et al. Their study showed elevated rate of lung cell proliferation related to high FoxM1 expression in patients exposed to sulfur mustard, a well known agent able to induce ROS [83].

Beyond the mechanisms described, the molecular basis of FoxM1 dysregulation has been also related to the capability of vitamin D receptor (VDR)/FoxM1 axis to affect cell stemness and to induce an invasive and metastatic phenotypes in pancreatic cancer. Indeed, the authors observed that VDR activation reduced the levels of FoxM1, inducing nuclear accumulation of *β*-catenin [84].

In prostate cancer (PCa), only few studies focused on the role of FoxM1; for instance, FoxM1 and its target CENPF, a structural protein of kinetochore, have been both proposed as critical drivers of PCa development and as prognostic markers of poor survival [85]. Concordantly, Lin et al. unveiled different miRNAs regulating FoxM1-CENPF axis taking advantage of miRNA expression profile available in Taylor dataset of prostate specimens (normal, localized, and metastatic tissues) [86]. Notably, since CENPF regulates several genes important for metastasis, including MMP2, MMP9, LOX, CXCR4, and CXCL12, dysregulation of the miRNA-COUP-TFII-FoxM1-CENPF axis can inhibit also PCa metastatization [86].

Overall, these considerations identified FoxM1 as a potential anticancer therapeutic target. Unfortunately, the druggability of FoxM1 remains a big challange because of the lack of substrate-binding pockets and hydrophobic surfaces [72, 87]. Several in vitro studies proposed RNA interference (RNAi) as a strategy to knockdown FoxM1, either alone or in combination with ROS inducers, in order to provoke ROS-mediated cell death [82]. Some studies reported that proteasome inhibitors, including bortezomib or thiostrepton, directly reduce both FoxM1 expression and its transcriptional activity with the same efficacy as that obtained by FoxM1 silencing [82, 88]. This latter approach is very promising, considering that bortezomib is already in clinical practice to treat multiple myeloma, and that RNAi treatment, so far, is not a reasonable therapeutic approach in patients [50, 82, 88]. Thus, bortezomib treatment has been proposed as effective therapeutic strategy in highly expressing FoxM1 solid tumor, also in association with ROS inducers.

## 6. Thioredoxin, Glutathione Peroxidase, and Superoxide Dismutase Families as Mediators of Carcinogenesis

Thioredoxin system, composed of thioredoxin reductase (TrxR), thioredoxin (Trx), and NADPH, senses and responds to oxidative stress and modulates the redox status by scavenging ROS and by regulating several redox enzymes and signaling proteins. Mammalian genomes encode two main Trx systems: Trx1 and Trx reductase (TrxR) 1, which together constitutes the cytosolic system; Trx2 and TrxR2, which are localized in mitochondria (a Trx3 isoform has been also reported, as a testis-specific form, mainly expressed in male germ cells and associated to reproductive disorders) [89]. Trx1 reducing power allows the transfer of two electrons from its dithiol motif to an acceptor, then the oxidized disulfide form of the enzyme is recycled to the dithiol form by TrxR1, thereby oxidizing one molecule of NADPH.

Interestingly, our analysis revealed that TXNRD1, the gene encoding TrxR1, is upregulated and correlates with bad prognosis in pancreatic, colon, HNSCC, lung, prostate, and breast cancers. Trx1 enzyme has been shown to regulate NF-*κ*B, playing opposite roles, depending to its intracellular localization: overexpression of Trx in cytoplasm reduced NF-*κ*B activity, blocking the degradation of the NF-*κ*B inhibitor I*κ*B; in the nucleus, Trx directly reduces the cysteine(s) of NF-*κ*B allowing the NF-*κ*B-dependent gene expression [90]. Following NF-*κ*B stimuli, such as UVB irradiation and TNF*α* treatment, Trx quickly translocates from the cytoplasm into the nucleus. Trx1 has also been reported as a secreted protein by normal and neoplastic cells [91], but not via exosomes [92]. Notably, Trx-increased secretion contributed to high ROS production in cisplatin-resistant lung tumors, both in vitro and in vivo [93].

Trx1 itself is regulated both by hypoxia and by oxidative stress conditions via binding of NRF2 to an antioxidant responsive element in the Trx promotor [94]. Moreover, Trx1 complex functions as a molecular switch turning the cellular redox state into kinase signaling. Thus, the system is able to regulate DNA synthesis, cell proliferation [95, 96], apoptosis, and transcription. In details, the reduced form of Trxs binds to apoptosis signal-regulating kinase 1 (ASK1) and inhibits its activity to prevent stress- and cytokine-induced apoptosis; when Trx is oxidized, it dissociates from ASK1 and apoptosis is stimulated [97–100]. The impact of Trx1 intracellular localization on its role may be taken into account especially in tumors (as colon and prostate) where a low expression of TXNRD1 correlates to poor patient outcome (as described in Figure 3()). In fact, although increased Trx1 protein expression has been associated to hypoxic regions of certain tumours, tumor grade and chemoresistence, for instance by scavenging ROS species generated by various anticancer agents [101, 102], its localization and activity have to be both taken into account. In prostate cancer, Shan and colleagues identified constitutive nuclear and transiently increased cytoplasmic Trx1 oxidation by androgen but decreased Trx1 activities with the progression of prostate cancer, despite high levels of Trx1 protein expression in cancer cells [103]. The role of TrxR1 in dysplastic transformation has been pointed out in human breast epithelial cells, triggered by chronic oxidative stress [104]. In addition, Trx1 has been proposed as serum biomarker for either early diagnosis or prognosis of breast cancer in association with CEA and CA15-3 [105]. In non-small-cell lung cancer, Trx1 is able to modulate transcription of cyclooxygenase-2 via hypoxia-inducible factor- (HIF-) 1*α* [106]. It is actually worth to mention that many human cancers have low levels of thioredoxin-binding protein-2 (TBP-2), a Trx regulator which is able to bind Trx, blocking its reducing activity. These mechanisms have been identified as druggable: histone deacetylase inhibitors (HDACi) have been demonstrated to upregulate TBP-2 in various transformed cells, associated with a decrease in Trx levels [102].

Recently, Park and colleagues observed that TrxR2 is a novel binding protein for ribonucleotide reductase small subunit p53R2, which is involved in nuclear and mitochondrial DNA replication and repair, stimulating the enzymatic activity of TrxR in vitro. Their findings also suggest that p53R2 acts as a positive regulator of TrxR2 activity in the mitochondria both under normal physiological conditions and during the cellular response to DNA damage [107].

Although STRING analyses highlighted glutathione peroxidases (GPx) as one of the main family involved in oxidative stress adaptation, we found high heterogeneity in the disregulation of GPx family members among the tumor types we have investigated (Figure 5). GPx reduces either free hydrogen peroxide to water or lipid hydroperoxides to their corresponding alcohols. So far, eight different isoforms of GPx, 1 to 8, have been identified in humans, carrying different affinities for their substrates and different localizations. GPx1, found in the cytoplasm of mammalian cells, is mainly able to target the hydrogen peroxide, while GPx4 showed high affinity for lipid hydroperoxides. GPx2 is an intestinal and extracellular enzyme, while GPx3 is extracellularly secreted [99].

GPx1 allelic loss or polymosphisms have been known for years to contribute to both lung [108] and breast cancers [109]. Interestingly, in HNSCC cancer, almost all the isoforms showed low expression (Figure 3). In agreement, a decrease in GPx activity accompanied by SOD and CAT decrease as well as higher levels of oxidative DNA damage was found in HNSCC patients compared to healthy donors [110].

An increase of both Trx and GSH metabolism is a mechanism widely implicated in the resistance of cancer cells to chemotherapy. Loss of TXNRD1 makes tumors highly susceptible to pharmacological GSH deprivation, and concomitant inhibition of both GSH and TxrR systems was recently proposed as an anticancer strategy [18, 111]. Recently, Rodman and colleagues demonstrated that depletion of GSH and inhibition of TrxR activity enhanced radiation responses in human breast cancer stem cells by a mechanism involving thiol-dependent oxidative stress [112]. Furthermore, Scarbrough and colleagues reported that simultaneous GSH/Trx inhibition sensitizes human breast and prostate cancer cells to 2DG + 17AAG-mediated killing [113].

Among the most important antioxidant enzymes, it is also important to highlight the role of SOD. SOD is able to convert the superoxide (O_2_^−^) radical into either oxygen (O_2_) or the less reactive hydrogen peroxide (H_2_O_2_) which can then be removed by CAT, GPx, or TPx. Among the three major families of SOD, those we single out in humans are the copper and zinc (Cu-Zn) SOD1, whose localization is in cytosol, nucleus, peroxisome, and intermembrane space of the mitochondria [114], the mitochondrial enzyme manganese SOD2 (MnSOD), and the (Cu-Zn) extracellular SOD3. SOD enzymes are able to exert a strong antioxidant activity. In a recent study, Elchuri and colleagues observed that mice deficient in CuZn SOD1 (which contributes to the majority of cellular SOD activity [115]) showed a reduced lifespan and increased incidence of neoplastic changes in the liver [116]. Conversely, it has been also observed by several authors that SOD1 overexpression makes tumor cells resistant to oxidative stress and chemotherapy [117]. Increased expression and activity of MnSOD has been correlated with cancer aggressiveness in several tumors and through different pathways [118]. Recently, dysregulation of MnSOD function has been linked to an acetylation-mediated impairment [119, 120] which triggers an increase in oxidative stress, leading to AKT activation via oxidative inactivation of PTEN [119]. MnSOD acetylation (and activity) is regulated by the deacetylase Sirt3, a mitochondrial fidelity protein. Interestinlgly, Zou et al. showed that loss of Sirt3 results in endocrine therapy resistance of human luminal B breast cancer [120]. In agreement, we and others demonstrated that HDAC inhibition increases MnSOD protein expression in both solid and haematological diseases [121, 122].

Overall, similar to FoxM1, the described antioxidant systems represent putative good targets to improve therapeutical oxidative stress-dependent strategies. In details, several recent efforts have focused on the targeting of Trx/TrxR system [123–130]. Moreover, increasing evidences on a putative key role of HDAC inhibitors in the modulation of these pathways may deserve further investigations. In this regard, our recent study on the effect of HDACi in regulating NRF2/Keap1 pathway is of interest, considering the interplay between this pathway and thioredoxin [7].

## 7. Oxidative Stress and Cancer Stem Cells

In the multitude of morphological, functional, and responsive cancer cells, a subset of the so-called “cancer stem cells” (CSC), carrying peculiar features, was identified almost ten years ago in solid cancers [131]. However, the name CSC is not referred to an origin from normal stem counterpart but rather represents a specific population that displays some exceptional properties normally attributed to stem cells. Specific features, like hierarchical differentiation, self-renewal, enhanced invasive capacity, metastatic proficiency, and tumorigenicity, make CSC critical for tumor initiation and growth [132], while CSC elevated apoptosis resistance, drug-efflux pumps, enhanced DNA repair efficiency, detoxification enzyme expression, and quiescence are all identified as prosurvival mechanisms associated with resistance to chemotherapy and tumor relapse [133].

Few studies reported the behavior of cancer stem cells in oxidative stress condition, but notably in contrast to their normal stem cell counterparts, cancer stem cells are characterized by increased ROS levels, reduced oxidative damage, and thus longer survival [134, 135]. For example, Im and colleagues showed that significantly higher ROS levels were observed in the supernatant of glioblastoma cells, grown in serum-free sphere medium, either in polystyrene-treated tissue culture plates or in nonadherent plates. Moreover, it has been also shown that ROS is a critical factor for maintaining stemness, regulating the expression of the transcription factor SOX-2 [136]. This can be due to a combination of mechanisms that arise in the tumor, such as modulation of (1) multiple antioxidative enzyme systems [137] or (2) redox-sensitive signaling pathways, as NRF2, NF-*κ*B, c-Jun, and HIFs, leading to the increased expression of antioxidant molecules [5].

The higher ROS levels in CSC could be associated with lower basal expression of ROS-scavenging systems, such as SODs, CAT, GPx, and TPx, compared to normal stem cells. In this regard, Yang et al. published those nonglioma stem cells which displayed significantly lower basal GPx1 expression and activity than glioma stem cells and that miR-153/NRF2/GPx1 pathway plays an important role in regulating radiosensitivity and stemness of glioma stem cells via ROS [138].

**Figure 6:**
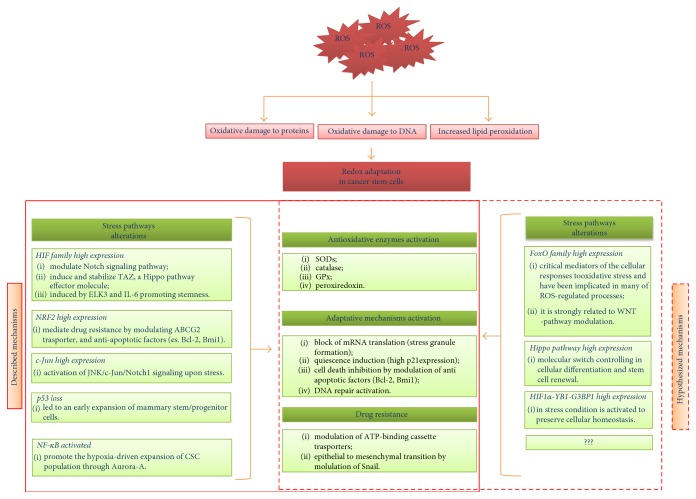
Redox stress in cancer stem cells. The persistent production of abnormally large amounts of ROS induced the mechanism of redox adaptation that, in turn, is translated in a various alteration in stress signaling. Here, we reported both known and hypothesized modulated pathways.

Due to the growing body of studies focused on the differential modulation of redox-sensitive signaling pathways (as summarized in Figure 6) in CSC subpopulation, compared to cancer cells or normal stem cells, in this paraghraph we discuss the relevance of the ROS-related pathways modulated in CSC phenotype.

In hypoxic environments, limited amount of oxygen leads to metabolic switches in both normal and malignant cells by HIFs. Paradoxically, recent studies have shown that CSC exhibit high HIF activity in normoxic environments and that HIF activity is critical in the maintenance of CSC as well as in the differentiation [139]. In agreement, Wang et al. found that overexpression of stem cell factor in hepatocellular carcinoma is regulated by hypoxic conditions through a selective HIF2*α*-dependent mechanism which promotes metastasis [140].

Several studies showed that HIF factors can enhance CSC population growth by modulating Notch signaling pathway in glioma [141], Hippo pathway through direct stabilization of TAZ in breast cancer [142], Ras-ERK-ELK3 in hepatocellular cancer, hypoxia-NOTCH1-SOX2 in ovarian cancer [143], and IL6-HIF1*α* in non-small-cell lung cancer [144]. Additionally, Yang et al. established that gastric CSC exhibited a marked increase in HIF1*α* expression and increased migration and invasion capabilities compared with the normoxic control upon hypoxia treatment. Also HIF-1*α* was responsible for activating EMT via increased expression of the transcription factor Snail in gastric CSC [145].

NF-*κ*B is also related to hypoxia and HIF1*α* induction. It has been shown that inhibition of NF*κ*B signaling promoted a significant reduction in the hypoxia-driven expansion of CD44^+^CD24^−/low^ CSC which was due to increased CD24 expression in breast cancer models [146]. Similarly, Aurora A kinase which can activate NF-*κ*B pathway has been found highly expressed in ovarian CSC [147].

NRF2 represents another antioxidant system involved in the maintenance of quiescence as well as in the determination of differentiation fate in normal stem cells, as described and reviewed by Ryoo et al. [148]. For example, NRF2-deficient mice showed defective stem cell function. Indeed, haematopoietic stem cell, derived from those mice, dispayed lower levels of prosurvival cytochines and exibited spontaneous apoptosis related to wild-type cells [149].

Recently, several studies showed that high levels of NRF2 are related to CSC survival and anticancer drug resistance in HNSCC, cervical, breast, and ovarian cancers [150–153]. Notably, it was reported that NRF2 overexpression is related to an induction of ATP-binding cassette trasporters and thus drug resistance mechanisms. Other described redox-signaling pathway implicated in redox regulation in CSC could be c-Jun and/or p53 and NF-*κ*B and FoxO family. In details, Chiche et al. showed that the loss of p53 in K5ΔN*β*cat (*β*cat activated) mice led to an early expansion of mammary stem/progenitor cells and accelerated the formation of triple-negative breast cancers. In particular, p53-deficient tumors expressed high levels of integrins and extracellular matrix components and were enriched in cancer stem cells [154].

Moreover, Xie et al. found that knockdown of JNK1 or JNK2 or treatment with JNK-IN-8, an adenosine triphosphate-competitive irreversible pan-JNK inhibitor, significantly reduced cell proliferation, the ALDH1+ and CD44+/CD24- CSC subpopulations, and mammosphere formation, indicating that JNK family promotes CSC self-renewal and maintenance in triple-negative breast cancer [155].

However, other factors could be implicated in CSC capability to adapt high level of intracellular ROS and would be very interesting to better define them as potential therapeutic targets, mostly because many anticancer drugs increase intracellular ROS levels.

In this regard, the transcription factors FoxO1, FoxO3a, and FoxO4 are critical mediators of the cellular responses to oxidative stress and have been implicated in many of ROS-regulated processes [156]. It is also known that FoxO competes with TCF for the same binding site of *β*-catenin and suppresses *β*-catenin-TCF signaling toward proliferation, thus attenuating WNT-mediated signaling activities. Also, FoxO factors reduce mitochondrial output to prevent excess ROS production through inhibition of c-Myc function and alter the hypoxia response [157].

Another candidate is the Hippo pathway, which acts as a molecular switch controlling in cellular differentiation and stem cell renewal but is also modulated in stress condition and is described as highly mutated in cancer. Lehtinen and colleagues elegantly demonstrated the activation of Mst1, a serine/threonine kinase activated in the Hippo cascade, upon oxidative stress induced by exposure to increasing concentrations of exogenous H_2_O_2_. This was accompanied by phosphorylation of the transcription factor FoxO3a at S207, thereby disrupting its association with 14-3-3 binding protein and leading to its nuclear localization and transcriptional activation of the BH3- only Bcl-2 protein, Bim, which triggered neuronal apoptosis [158].

One of the first mechanisms modulated upon stress condition is messenger RNA translation, likely as a mean to limit energy demanding protein synthesis, leading to stress granule (SG) formation in cancer cells. Many evidences suggest that altered mRNA translational control is a critical factor in cancer progression, and in this regard, a new axis has been described. In details, Somasekharan et al. showed that under stress condition, a YB1, nuclease-sensitive element-binding protein 1, facilitates tumor metastasis through two mechanisms: first, it directly binds to HIF1*α* that drives stress adaptation and metastatic capacity in vivo; *s*econd, YB1 mediates formation of cytosolic SGs through translational activation of G3BP1, a SG nucleator [159]. Accumulating evidences suggest that SG formation is protective against stress-induced cell damage and death [160], and few studies suggested SG implication in cancer biology [161, 162].

## 8. Conclusions and Future Perspectives

The idea that the oxidative stress modulation has a crucial role in cancer cells to promote proliferation, adaptation, and resistance to therapy is now widely accepted [7, 12, 13]. Thus, modulating redox regulatory mechanisms represents an attractive therapeutic strategy. However, to date, the oxidative stress-related therapeutic strategies evaluated in preclinical and clinical studies did not produce homogenous results, due to several variables associated to ROS generation and redox adaptation mechanisms.

Furthermore, the identification of tumor-type specific oxidative stress gene profiles and how they could predict prognosis still represent critical challenges. Thanks to the increasing availability of cancer gene expression profile, mutation, epigenetic, and survival data from the TGCA dataset, it was possible to use bioinformatics to screen the role of oxidative stress genes from a publicly available signature in large cohorts of several solid cancer patients [52, 53].

The TCGA database provides correlative evidences suggesting the involvement of the FoxM1, thioredoxin, superoxide-dismutase, and glutathione pathways as principally and commonly modulated in breast, lung, HNSCC, pancreatic, prostate, and colon cancers. The differential expression levels of each gene observed in different settings revealed a precise spatial context where redox alterations may promote genome instability or redox adaptation.

For this reason, tumors should be classified into subclasses based on different oxidative stress alterations occurring in redox homeostasis genes, to guarantee the development of precision medicine-based approaches in selected subgroups of cancer patients. Further mechanistic studies are needed to identify either new compounds or molecules to be repositioned, in order to target the described redox pathways.

## Supplementary Material

Supplementary Figure S1. Kaplan Meier curves showing the survival in the case of high and low expression of DUSP1, EPHX2, NUDT1, RNF7 and SEPP1 in solid cancers patients. Supplementary Figure S2 STRING analysis of modulated oxidative stress genes in six different type of cancer. Supplementary Figure S3 STRING analysis of oxidative stress genes in six different type of cancer: high vs low network.
