# Supplementary material for: Oxidative Stress Gene Expression Profile Correlates with Cancer Patient Poor Prognosis: Identification of Crucial Pathways Might Select Novel Therapeutic Approaches
Source: Oxid Med Cell Longev. 2017 Jul 9;2017:2597581. doi: 10.1155/2017/2597581 (PMC5523271; doi:10.1155/2017/2597581)
Supplement: Supplementary file 1 — Supplementary Figure S1. Kaplan Meier curves showing the survival in the case of high and low expression of DUSP1, EPHX2, NUDT1, RNF7 and SEPP1 in solid cancers patients. Supplementary Figure S2 STRING analysis of modulated oxidative stress genes in six different type of cancer. Supplementary Figure S3 STRING analysis of oxidative stress genes in six different type of cancer: high vs low network. [file 2597581.f1.docx]

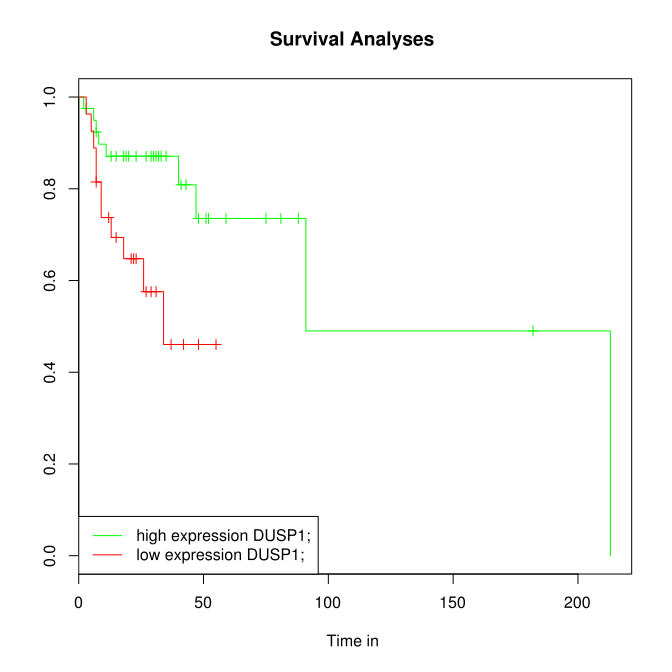

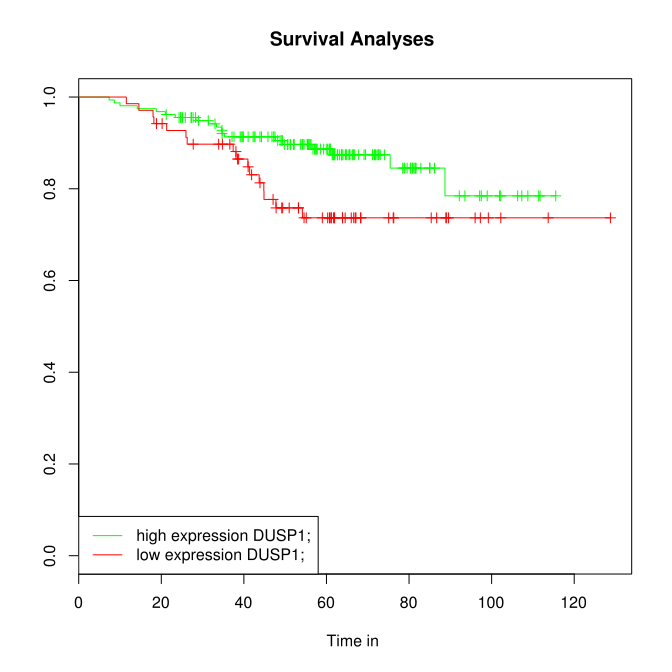

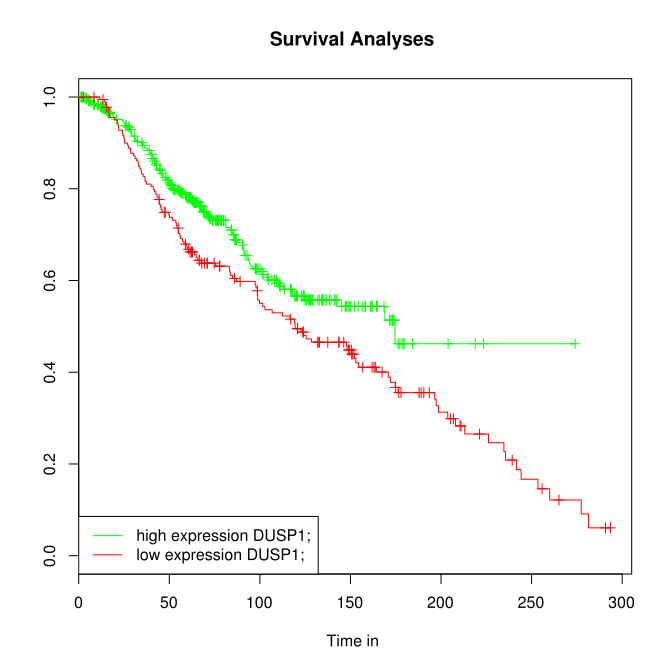

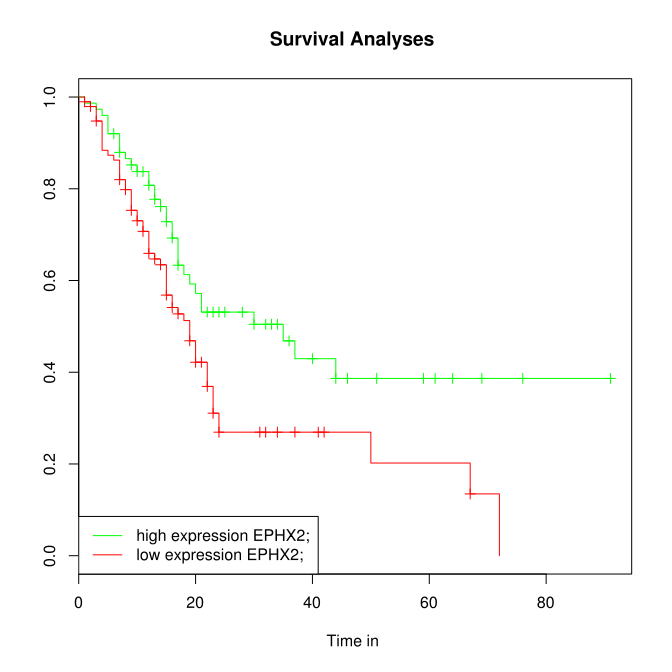

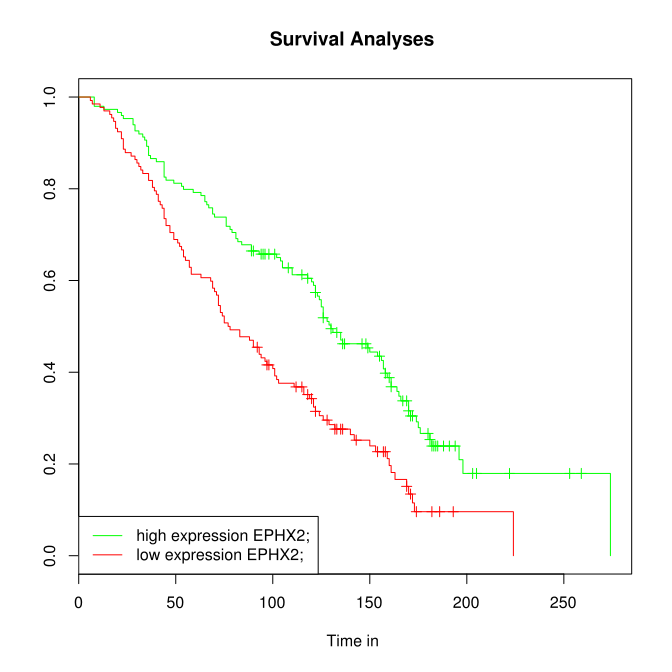

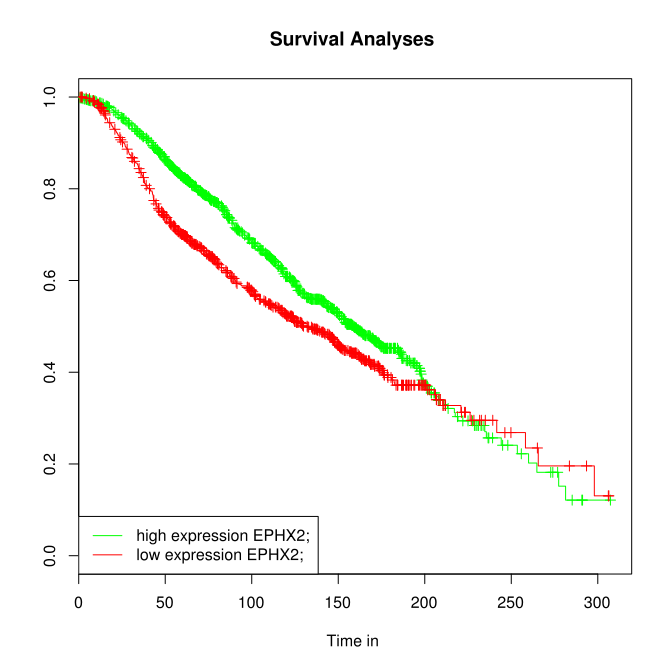

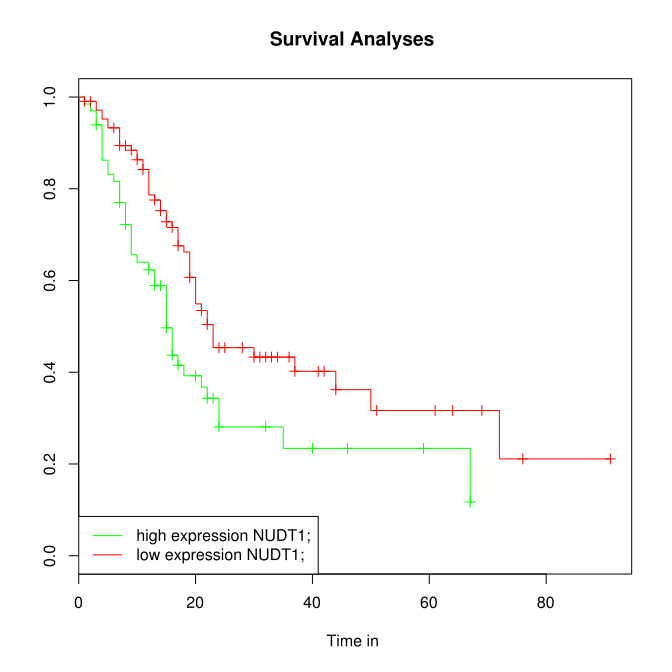

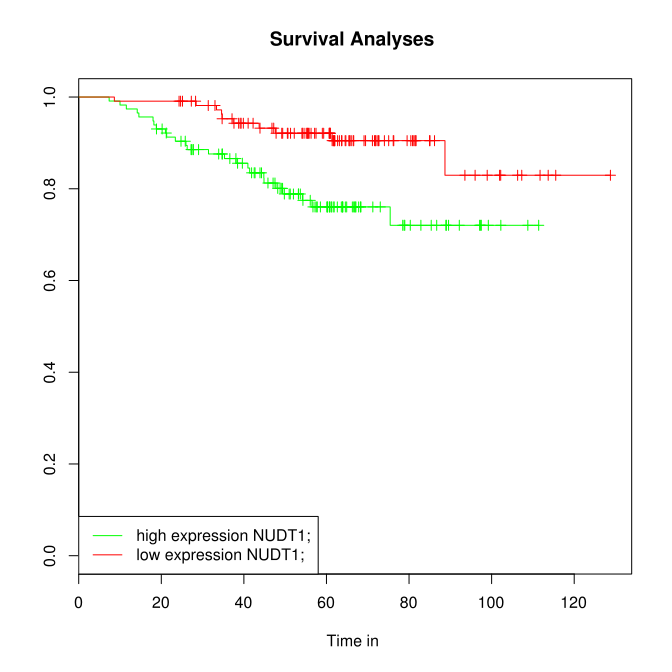

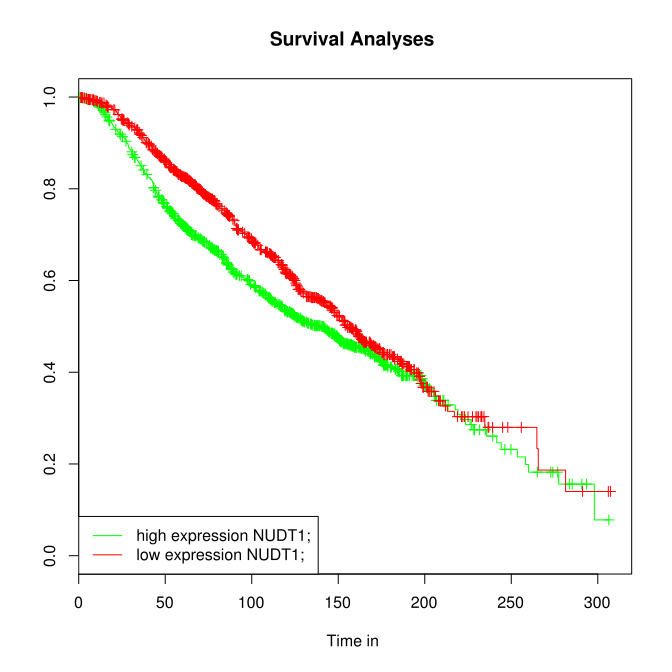

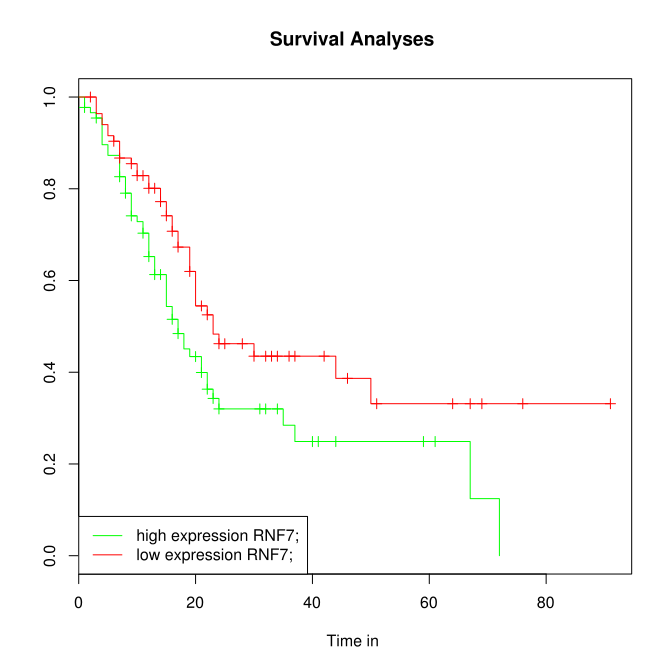

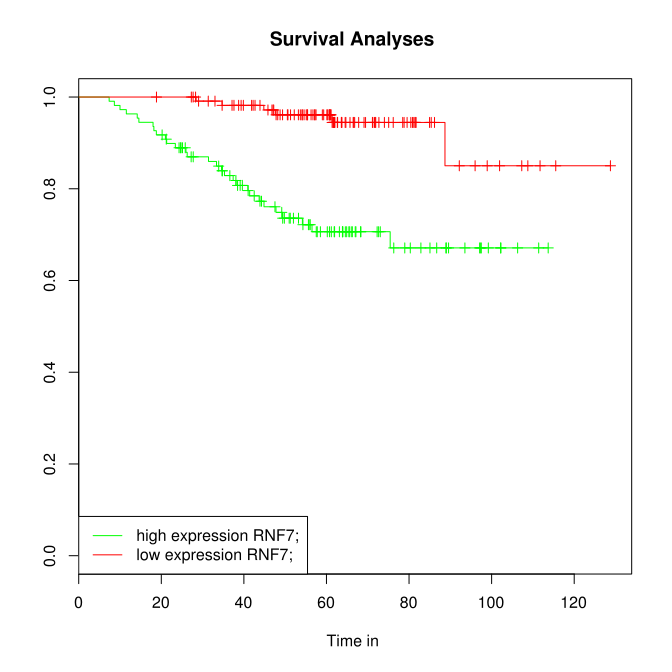

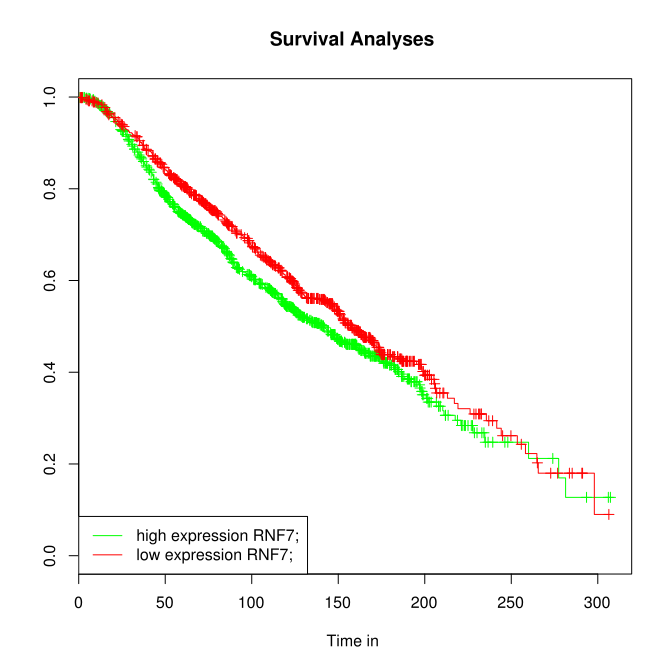

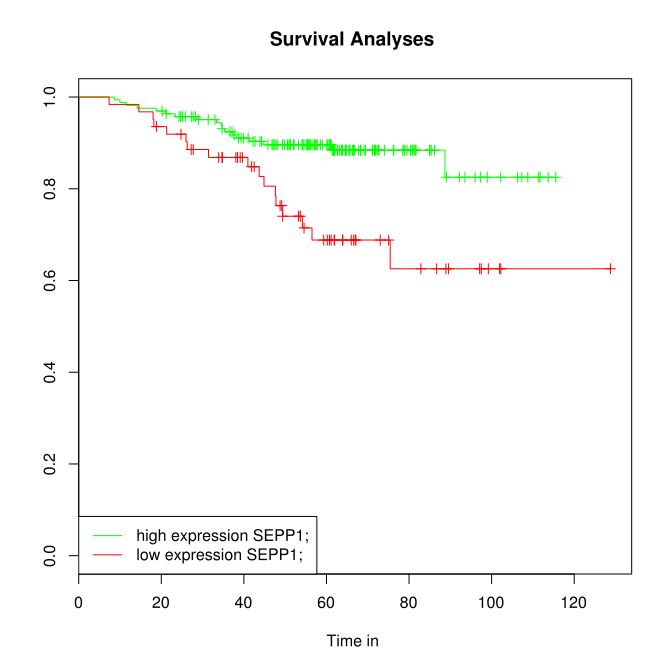

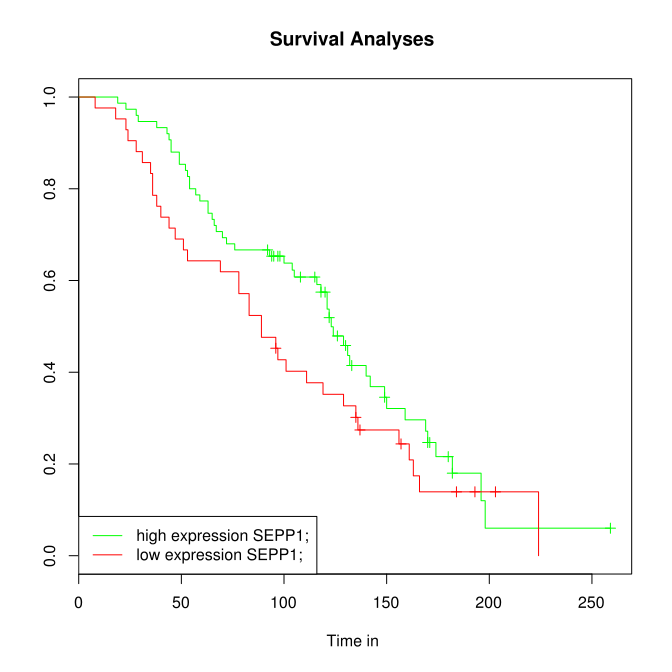

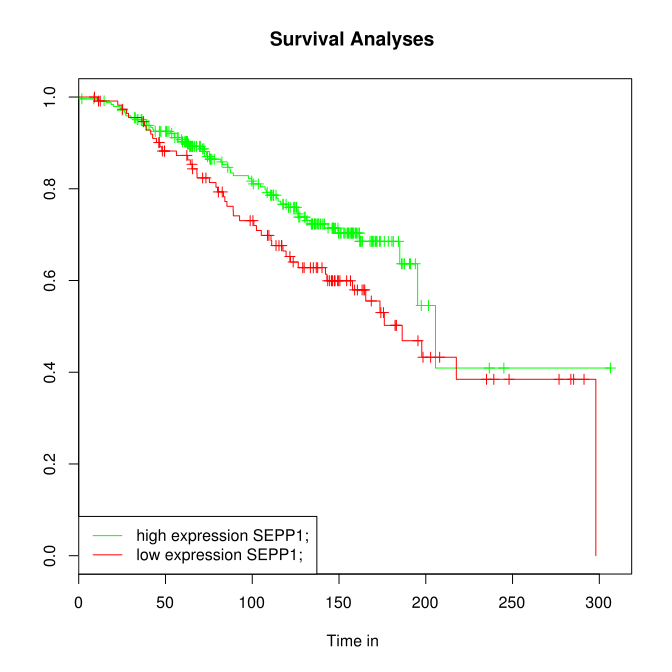


**DUSP1**

Head & Neck

(TCGA_HNSCC)

Lung

(GSE31210)

Breast cancer

(METABRIC)

Pancreas

(TCGA_PAAD)

Prostate

(TCGA_PRAD)

**EPHX2**

Breast cancer

(METABRIC)

**NUDT1**

Pancreas

(TCGA_PAAD)

Lung

(GSE31210)

Breast cancer

(METABRIC)

**RNF7**

Pancreas

(TCGA_PAAD)

Lung

(GSE31210)

Breast cancer

(METABRIC)

**SEPP1**

Prostate

(TCGA_PRAD)

Breast cancer

(METABRIC)

Lung

(GSE31210)

Supplementary Figure S1
