# Supplementary material for: Oxidative Stress Gene Expression Profile Correlates with Cancer Patient Poor Prognosis: Identification of Crucial Pathways Might Select Novel Therapeutic Approaches
Source: Oxid Med Cell Longev. 2017 Jul 9;2017:2597581. doi: 10.1155/2017/2597581 (PMC5523271; doi:10.1155/2017/2597581)
Supplement: Supplementary file 2 [file 2597581.f2.docx]

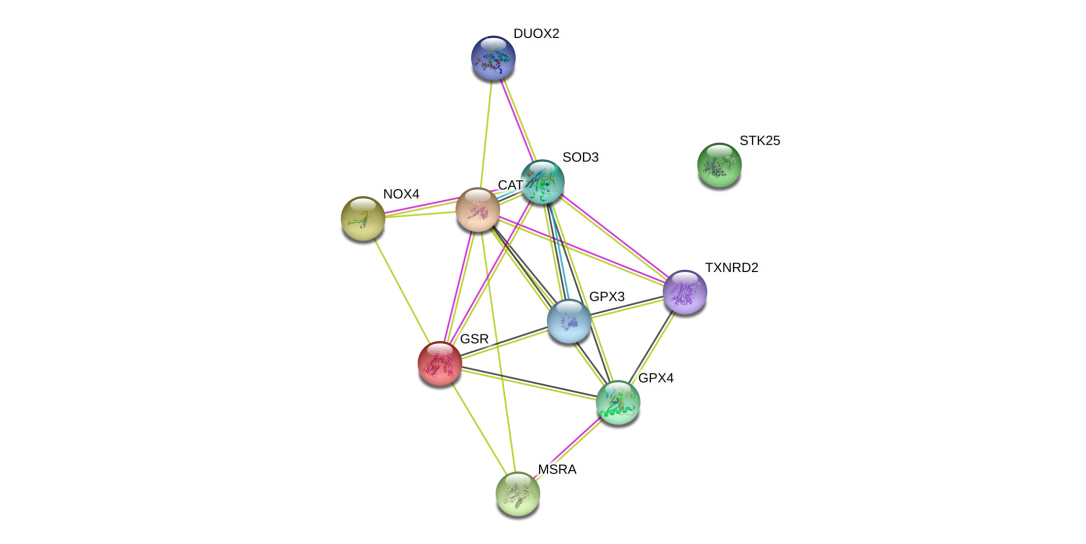

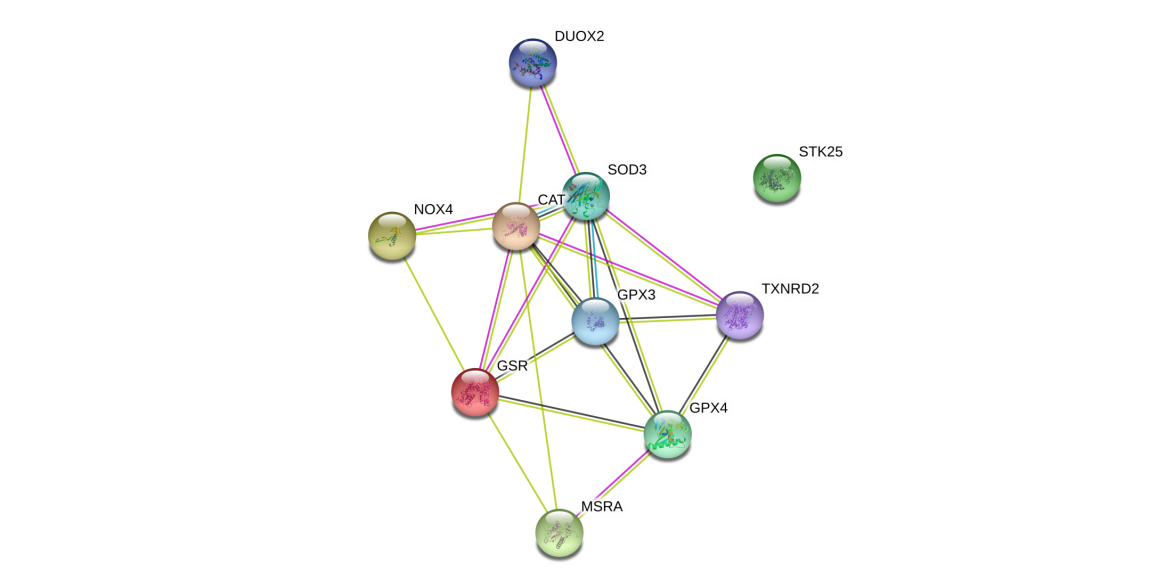

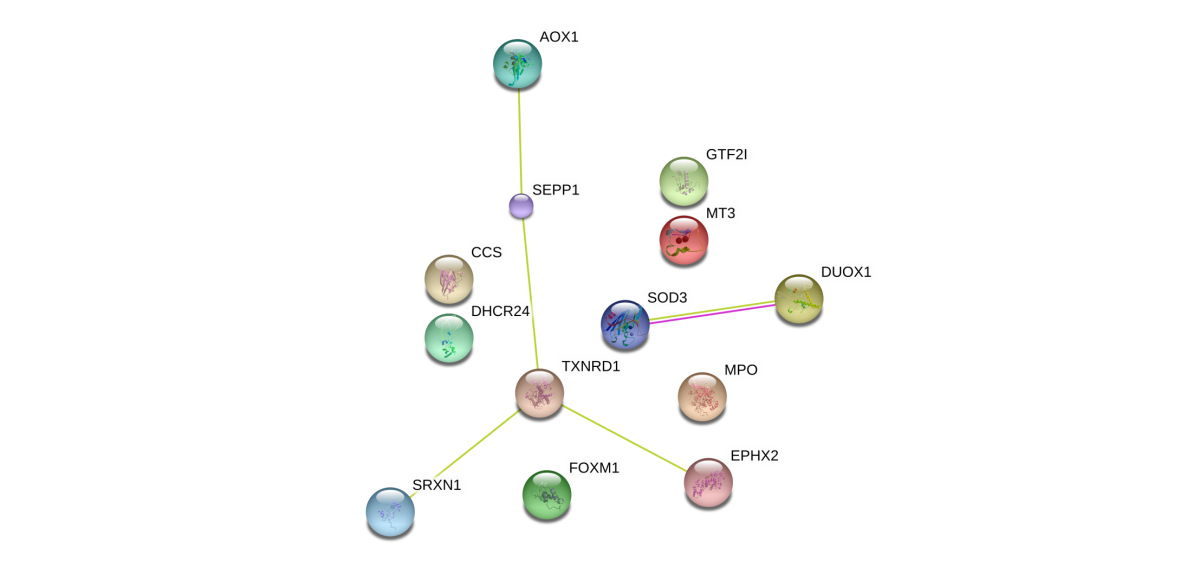

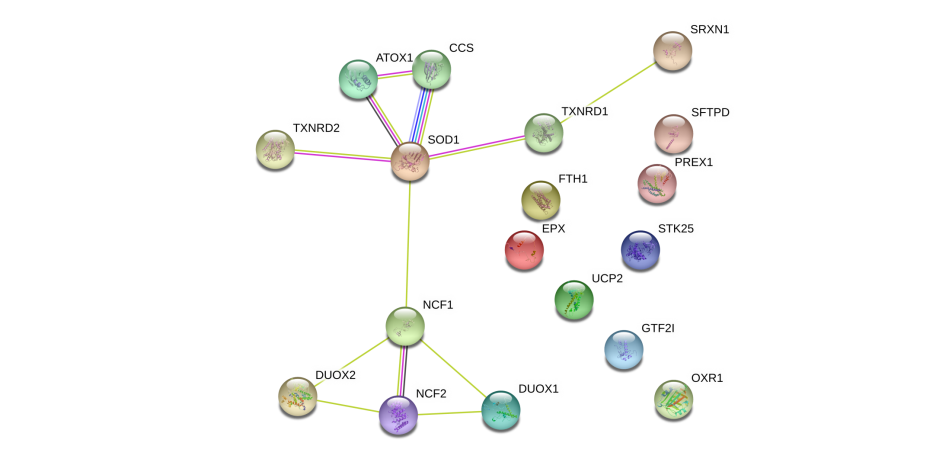

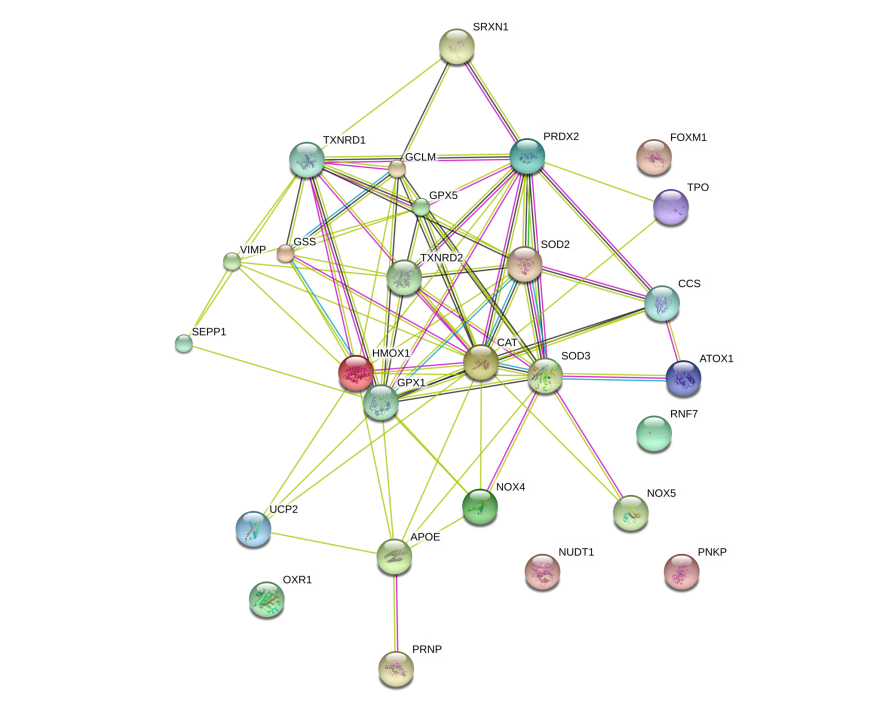

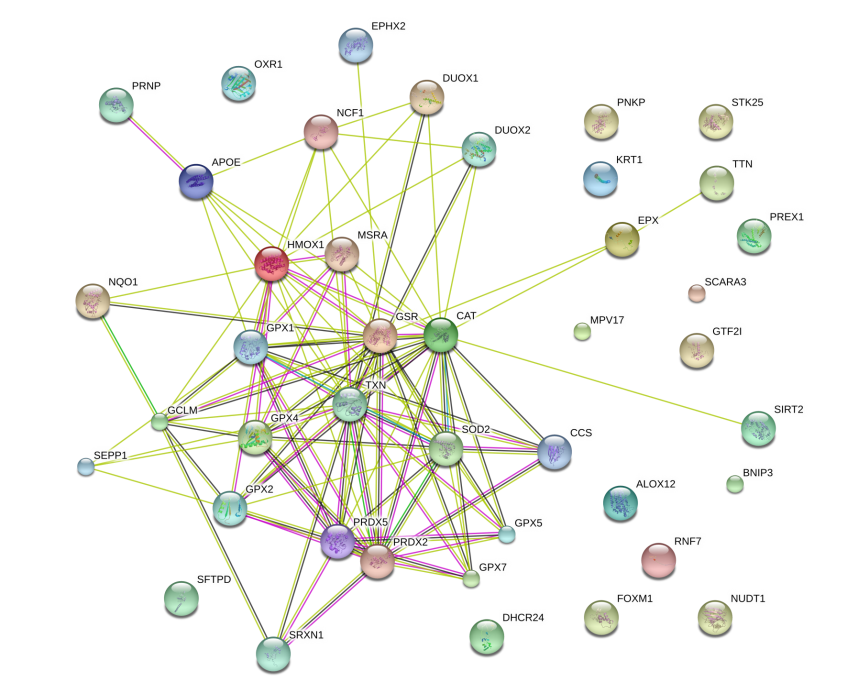


**Colon 12 genes**

**Pancreas 14 genes**

**Prostate 13 genes**

**H&N 20 genes**

**Lung 29 genes**

**Breast 43 genes**

Supplementary Figure S2
