# Supplementary figures and images for: Oxidative Stress Gene Expression Profile Correlates with Cancer Patient Poor Prognosis: Identification of Crucial Pathways Might Select Novel Therapeutic Approaches
Source: Oxid Med Cell Longev. 2017 Jul 9;2017:2597581. doi: 10.1155/2017/2597581 (PMC5523271; doi:10.1155/2017/2597581)

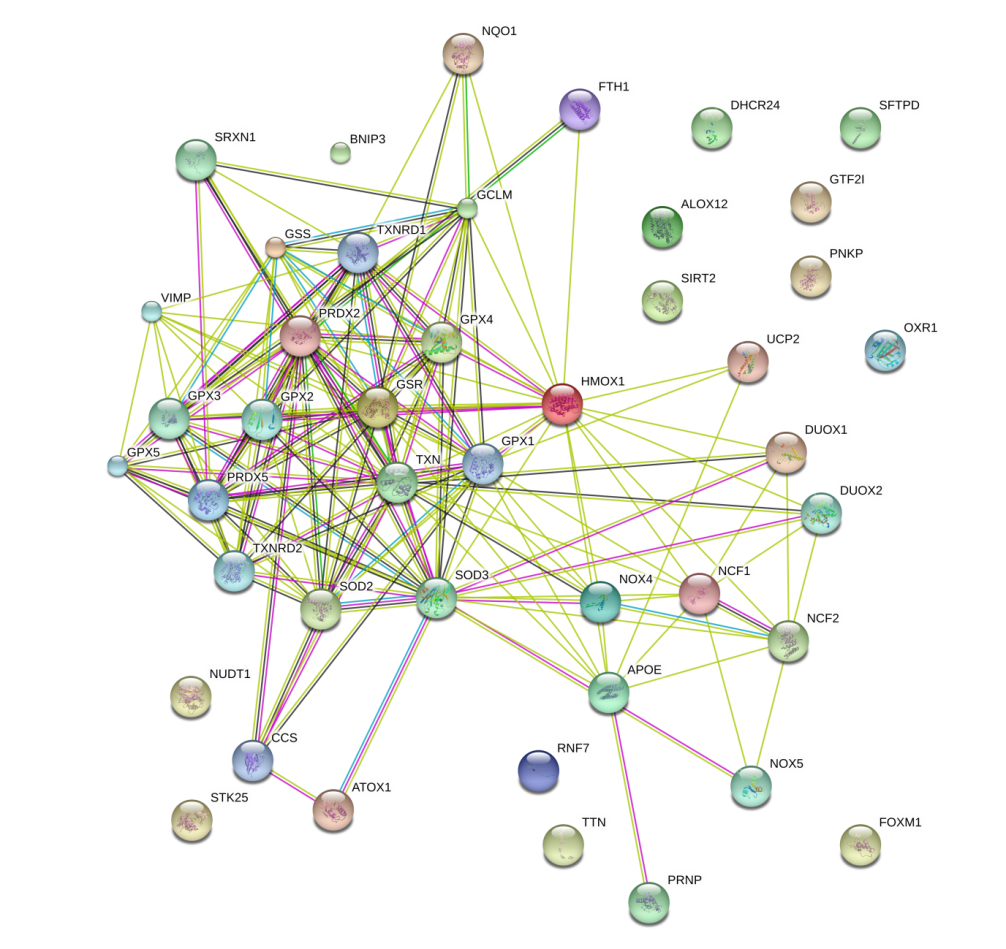

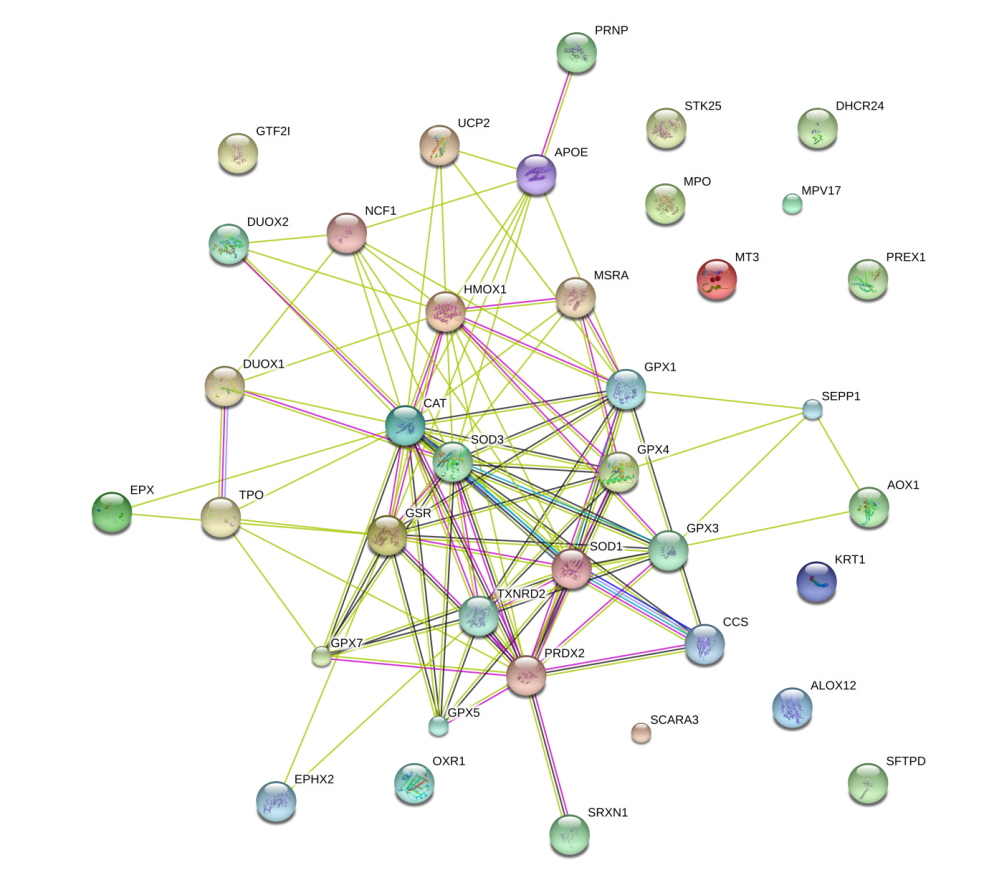


**High: 44 genes**

**Low: 38 genes**

Supplementary Figure S3

Supplement: Supplementary file 3 [file 2597581.f3.docx]
